# Prevalence and allele frequency of Congenital Colour Vision Deficiency (CCVD) among students at Hawassa University, Ethiopia

**DOI:** 10.1186/s42506-020-00037-y

**Published:** 2020-03-20

**Authors:** Reta Gutema Mitiku, Bekele Serbessa Tolera, Zelalem Gebremariam Tolesa

**Affiliations:** 1 Department of Biology, Dambi Dollo University, Dambi Dollo, Ethiopia; 2 Department of Biology, Wollega University, Nekemte, Ethiopia; 3 Department of Biology, Hawassa University, Hawassa, Ethiopia

**Keywords:** Colour vision deficiency, Deutan, Protan, Red-green deficiency, X-linked recessive

## Abstract

**Background:**

The prevalence of congenital colour vision deficiency (CCVD) varies from race to race and differs in different geographic regions. Colour vision deficiency or colour blindness, is the inability or decreased ability of discriminating certain colour combinations and colour differences under normal lighting conditions. This study aimed to determine the prevalence of congenital colour vision deficiency among students at Hawassa University.

**Methods:**

A cross-sectional survey was employed involving 4004 students (females = 1171 and males = 2833) from four campuses, namely, Institutes of Technology, College of Health Science and Medicine, College of Agriculture and Main Campus. The Ishihara pseudo-isochromatic 24 plate edition was used to test the colour vision of students under natural day light condition.

**Results:**

The prevalence of CCVD in the present study was 2.85%. A hundred and six (3.75%) males and eight (0.68%) females were affected with congenital colour vision deficiency. The frequencies of achromacy, deutan and protan in male subjects were 4 (0.14%), 82 (2.89%), and 24 (0.85%), respectively. Deutan was highest among students of Amhara ethnic origin (38, 2.51%), but the frequency of protan was highest amongst Oromo students (10, 0.8%).

**Conclusion and recommendations:**

The overall prevalence of CCVD found in the present study was lower compared to the previous studies done in Ethiopia. There was clear variation in the prevalence of colour vision deficiency among students of various ethnic groups. Proper screening, education and counseling are needed to minimize impacts of CCVD in the country, and can also be beneficial for the affected subject in tackling difficulties in everyday work and for proper choice of future profession.

## Introduction

Normal colour vision in humans is mediated by the three classes of cone photoreceptors (trichromatic colour vision): the blue or shortwave-sensitive (S), the green or middle-wave-sensitive (M), and red or long-wave-sensitive (L) [1]. Colour vision deficiency (CVD), is the inability or decreased ability to discriminate certain colour combinations and colour differences under normal lighting conditions. Most colour vision defects are congenital and permanent, but rarely it may be acquired [2, 3]. Red-green defects (Protan and Deutan) show the highest prevalence in the general population. Red-green colour blindness, is genetically determined by X-linked recessive gene [4]. The genes causing red-green defects are localized to the long arm of the X chromosome at Xq28 [5–8], whereas the blue pigment gene is located on an autosome, chromosome 7 at 7q32 [7–10]. Green pigment genes vary in number among colour-normal individuals and, together with a single red pigment gene, are proposed to reside in a head-to-tail tandem array within the X chromosome [9]. Acquired CVD may be caused due to factors such as damage to the optic nerves, metabolic disorders (e.g., diabetes), eye diseases (e.g., glaucoma, macular degeneration), chronic illness (e.g., sickle cell anemia), drug overdose (e.g., barbiturates, digoxin, anti-tubercular drugs) [11].

The prevalence of colour congenital blindness varies from race to race and differs in different geographic areas. For example, the prevalence is 3.36% in Saudi Arabia [12], 5.28 %, in Manipur (India) [13], 3.28% in Shekhan City/Kurdistan region (Iraq) [14], 4.10% in Welkite town (central Ethiopia) [15] and 2.50% in Bhopal (India) [16]. For the gene being X-linked, the frequency of CCVDs varies between males and females and it is usually more frequent in males. The prevalence of CCVD in males is 5.58% in Nepal [17], 8.47% in Erbil City (Iraq) [18] and 4.2 % in Bhopal (India) [16]. The prevalence of CCVDs varies among females from population to population, for example, 0.46 % among the Basque population (Spain) [19], 0.75% in Saudi Arabia [12], and 0.93% in Qazvin (Iran) [20].

People with defective colour vision are at a disadvantage, especially for employment in professions like pilots, drivers, in defense services and in technical fields like engineering and the medical profession [17, 21]. In addition, in some fields of studies such as electrical engineering, agriculture, laboratory, forensic sciences, chemical engineering, soil engineering and architectural engineering normal colour vision is important [2, 13, 22]. In Ethiopia, there are a limited number of studies done to assess the prevalence of congenital colour vision deficiency and its potential professional impacts [23–28]. The objective of the present study was to assess the prevalence and allele frequencies of red-green colour vision defects among students at Hawassa University, Ethiopia.

## Materials and methods

### Study area

The study was conducted at Hawassa University focusing on undergraduate and postgraduate students from four campuses, namely; Institute of Technology, College of Health Science and Medicine, College of Agriculture and the Main campus.

### Research design and study subjects

A descriptive cross-sectional survey was conducted to determine the prevalence of congenital colour vision deficiency. The inclusion criteria were Ethiopian students, above 18 years old, with the normal eye condition and who gave their consent. In this study, 4020 study subjects participated, but only 4004 individuals (i.e. females = 1171 and males = 2833) were included for subsequent analysis as the remaining 16 subjects had incomplete data. Furthermore, study subjects were from 35 ethnic groups.

### Congenital colour vision deficiency test

The Ishihara pseudo-isochromatic plates were used to assess the colour vision of students under natural day light condition. The numbered plates of the Ishihara chart were used and the abnormalities were judged following Ishihara’s recommendation [29]. The colour vision testing plates were held at 75 cm from the subject and tilted at right angles to the line of vision. The study subject was asked to read the numbers seen in the test plates 1 to 17. An assessment of the reading of plates 1 to 15 determines the normality or defectiveness of colour vision. If 13 or more plates are read correctly, the colour vision is regarded as normal. If only 9 or fewer plates are read correctly, the colour vision was regarded as red-green deficient. Achromacy subjects read correctly plate one only. The plates 16 and 17 are used to differentiate deutan and protan types of colour vision efficiency.

### Statistical analysis

Statistical analyses were conducted using SPSS version 20 [30]. The Chi-square (χ^2^) test was done to determine whether there was a significant difference between sexes and between ethnic groups.

### Allele frequency analysis

Assuming that the populations are non-consanguineous, the frequencies of the normal allele (p), deutan allele (q) and protan allele (s) for colour blind subjects were calculated based on the Hardy–Weinberg law using the gene counting method as was done by Shah et al. [13]. The expected allele frequencies of females were calculated based on the affected males’ allele frequencies but the actual allele frequencies of females were calculated based on the affected females.

## Results

### Phenotypic frequency of congenital colour vision deficiency

A total of 4004 study subjects took part in this study out of which 1171 (29.25%) were females and 2833 (70.75%) were males. The age of the study subjects ranged from 18 to 47 years with a mean of 21.25 ± 2.642 years. Of the total study subjects, 114 (2.85%; 95% CI: 2.33 to 3.37) had congenital colour vision deficiency, including 106 males and 8 females (Table 1). The prevalence

**Table 1:** The prevalence of congenital colour vision deficiency (CCVD) by gender and ethnic groups, Hawassa University, Ethiopia

	Combined			Male			Female		
Ethnic groups	Normal (%)	Color blind (%)	Total	Normal (%)	Color blind (%)	Total	Normal (%)	Color blind (%)	Total
Amhara	1465(96.96)	46 (3.04)	1511	1024(95.88)	44(4.12)	1068	441(99.55)	2(0.45)	443
Oromo	1109(96.94)	35 (3.06)	1144	732(96.06)	30(3.94)	762	377(98.69)	5(1.31)	382
Others†	1312(97.55)	33 (2.45)	1345	967(96.80)	32(3.20)	999	345(99.71)	1(0.29)	346
Total	3886(97.15)	114(2.85)	4000	2723(96.25)	106(3.75)	2829	1163(99.32)	8(0.68)	1171

^†^Afar (7), Agew (4), Alle (1), Antro (3), Anyuak (4), Arri (3), Awi (3), Bench (7), Burji (1), Dawuro (21), Dorze (2), Gamo (105), Gedeo (5), Gumuz (3), Gurage (182), Hadya (93), Halaba (13), Hamar (1), Harari (1), Kafa (14), Kambata (104), Konso (4), Kore (2), Kucha (3), Nyangatom (2), Shakacho (3), Shinasha (3), Sidama (306),Silte (39), Somali (20), Tegaru (193), Wolaita (191), and Yem (3) ethnic groups. The numbers in the brackets are the percentage of sample size subjects from the respective ethnic groups

of colour blindness in male and female students was 3.75% (95% CI: 3.05 to 4.45) and 0.68%, respectively.

In the present study, the subjects were from 35 different ethnic groups (Table 1). As the sample size of the majority of ethnic groups was small (except that of Amhara and Oromo), we had combined them together to have a balanced sample size. Colour blindness was only recorded among subjects from 10 ethnic origins, namely; Amhara, Gurage, Halaba, Kafa, Kambata, Oromo, Sidama, Somali, Tegaru and Wolaita. Deutan was highest amongst students of Amhara ethnic origin 38 (2.51%), but the frequency of protan was highest amongst Oromo students 10 (0.87%). Nonetheless, the prevalences of deutan and protan were low in the other ethnic groups combined. The frequencies of anchromacy, deutan and protan were 4/0, 82/6 and 24/2, in males/females respectively (Table 2). The frequency of deutan and protan among females of the affected students was higher in Oromo ethnic origin. In this study, total colour blindness was not observed in females (Table 2).

**Table 2:** Phenotypic frequency of achromacy and the different types of CCVD among male and female students of various ethnic groups in Hawassa University, Ethiopia

Ethnic groups	Male				Female			
	Achromacy (%)	Deutan (%)	Protan (%)	Total	Achromacy (%)	Deutan (%)	Protan (%)	Total
Amhara	2 (0.19)	36 (3.36)	8 (0.75)	1070	0 (0.00)	2 (0.45)	0 (0.00)	443
Oromo	1 (0.13)	22 (2.88)	8 (1.05)	763	0 (0.00)	3 (0.79)	2 (0.52)	382
Others	1 (0.10)	24 (2.40)	8 (0.80)	1000	0 (0.00)	1 (0.29)	0 (0.00)	346
Total	4 (0.14)	82(2.89)	24 (0.85)	2833	0 (0.00)	6 (0.51)	2 (0.17)	1171

### Allelic and genotypic frequency of CCVD among ethnic groups

Since males are hemizygous (i.e. having a single X chromosome), the frequency of the CCVD allele in them would be the same as the proportion of colour blindness (e.g. Amhara (44/1068 = 0.04), Oromo (30/762 = 0.04) and the other ethnic groups (32/999 = 0.03). Similarly, the observed (actual) frequencies in females were 0.005, 0.013 and 0.003 in Amhara, Oromo and other ethnic groups, respectively. Based on the frequencies of CCVD allele in males of the respective ethnic groups, the expected frequencies in females were (0.04^2^ = 0.0016) in Amhara and Oromo and (0.03^2^ = 0.0009) in the other ethnic groups combined. In this regard, the expected frequency of CCVD among female students of Oromo ethnic origin was 0.0016 and lower compared to the observed frequency (0.013). The frequency of heterozygote was higher (8.00%) in Amhara and Oromo but lower among study subjects in other ethnic groups combined (Table 3).

**Table 3:** The frequencies of normal, heterozygote, double heterozygote and colour blind male and female subjects

Ethnic group	Gender								
	Male				Female				
	Normal	Deutan	Protan	Total	Deutan	Protan	Heterozygote	Double heterozygote	Colour blind
Amhara	1024	36	8	1068	*q*^2^ = 0.001	*s*^2^ = 0.0001	2 (*pq* + *ps*) = 0.08	2*qs* = 0.001	*q*^2^ + *s*^2^ = 0.0011
	*p* = 0.96	*q* = 0.03	*s* = 0.01						
Oromo	732	22	8	762					
	*p* = 0.96	*q* = 0.03	*s* = 0.01		*q*^2^ = 0.001	*s*^2^ = 0.0001	2 (*pq* + *ps*) = 0.08	2*qs* = 0.001	*q*^2^ + *s*^2^ = 0.0011
Other	967	24	8	999					
	*p* = 0.97	*q* = 0.02	*s* = 0.01		*q*^2^ = 0.0004	*s*^2^ = 0.0001	2 (*pq* + *ps*) = 0.06	2*qs* = 0.0004	*q*^2^ + *s*^2^ = 0.001
Total	2723	82	24	2829					
	*p* = 0.96	*q* = 0.03	*s* = 0.01		*q*^2^ = 0.001	*s*^2^ = 0.0001	2 (*pq* + *ps*) = 0.08	2*qs* = 0.001	*q*^2^ + *s*^2^ = 0.0011

## Discussion

The overall prevalence of CCVD in the present study was lower compared to the previous studies such as in Addis Ababa (Ethiopia), 4.52%, by Abebe and Wondimkun [24], in Saudi Arabia, 3.36%, by Oriowo and Alotaibi [12], in Manipur (India), 5.28 %, by Shah et al [13], in Shekhan City/Kurdistan region (Iraq), 3.28%, by Abdulrahman [14], in Welkite town (central Ethiopia), 4.10%, by Woldeamanuel and Geta [15] but higher than a report in Bhopal (India), 2.50% by Gupta et al. [16]. The lower prevalence of CCVD in the present study relative to other studies conducted in Ethiopia [24, 25], could be due to the larger sample size of the current study. In this study, the majority of students were unaware about colour vision deficiency disease, except students from Health Science and Medicine.

The prevalence of CCVD recorded in males in the present study (3.75%) was similar to the reports in western Nepal (Pokhara, 3.80%) [31] and in Rajasthan (India, 3.20%) [32]. Yet the frequency of CCVD recorded amongst males in this study was low relative to investigations in Tehran (8.20%) [33], in Denmark (8.67%) [34], in Nepal (5.58%) [17], in Erbil City (Iraq, 8.47%) [18] and in Bhopal (India, 4.20 %) [16]. However, the prevalence of the CCVD (males) in the present study was higher than that of India (2.30%) [35] and in Qazvin (Iran, 2.56%) [20].

Several studies indicated that the prevalence of CCVD in females is usually lower as compared to that of males (e.g., [12, 13, 23, 31]). The prevalence of CCVD amongst females in the present study was higher than in Italy (0.10%) [36], in northern Ethiopia (0.20%) [23], but comparable to that of Saudi Arabia (0.75%) [12] and in Qazvin(Iran, 0.93%) [20]. A relatively higher frequency of CCVD in females is reported by couples of studies, for examples, in the Punjab city of India, (1.10%) [37] and Erbil City (Iraq, 1.37 %) [18]. However, studies conducted in Libya [35], in western Nepal (Pokhara) [31], in Rajasthan (India) [32] reported no cases of CCVD amongst female subjects. On the contrary, studies done in Faisalabad (Pakistan) [38] and in Kolkata (West Bengal/India) [39] reported a higher prevalence of CCVD in females compared to males. The higher prevalence amongst males as compared to females indicates the genetic causation of the disorder [4–10]. Congenital colour vision deficiency is genetically determined by X-linked recessive inheritance and thus occurs in males but is transmitted via females and about 8.0% of all women are carriers [40].

The prevalence of achromacy in this study was comparable with Abeshge district (central Ethiopia, 0.19%) [25] and in Ugep (Nigeria, 0.20%) [41]. On the other hand, the prevalence of deutan in males (2.89%) was lower compared to the northwest Ethiopia (3.20 %) [23], but similar to Abeshge district (2.89%) [25].

The prevalence of CCVD amongst students of Amhara (3.04%) and Oromo (3.06%) ethnic was lower compared to some ethnic groups in Nepal (e.g. 5.00% in Brahman, 5.10% in Gurung, 9.10% in Newar and 14.30% in Darji) but higher relative to other ethnic groups (e.g. 2.8% in Chhetri and 2.1% in Magar) [31]. Furthermore, the prevalence of CCVD in males (3.75%) and females (0.68%) in the present study was lower compared to various ethnic groups in Iraq (e.g. 8.45%/1.20% (males/females) in Kurd, 9.44%/2.24% in Arabs, 8.52%/1.56% in Turkman, and 7.40%/1.05% in Kldan) [18]. The lower frequencies of various types of CCVD in the present study compared to Iraq [18] and India [13], could be due to the common practices of consanguineous marriage in countries in the middle east and southeast Asia [12, 13, 18, 31, 35]. In this regards, in Ethiopia consanguineous marriage is rare as it is not promoted because of cultural, religious and legal factors. However, a couple other factors contribute for the difference in the prevalence of CCVD between populations and geographic regions, in addition to the degree of consanguineous marriage (e.g. population movements, the molecular structure of gene on the X chromosome, natural selection). For example, increasing migration of people within a country and across countries (e.g. in Saudi Arabia [12], in India [13]), might indirectly lead to increase in the rate of exogamous marriages that could contribute increase in the prevalence of CCVD. It is also suggested that increase incidence of colour blindness amongst the Caucasians may be due to difference in the molecular pattern of X chromosome of colour vision genes. Furthermore, it was observed that overall frequency of colour vision defects has been observed quite low amongst scheduled tribe groups (traditionally food gatherers and hunters and later occupied in shifting cultivation and as agricultural laborers) from all the zones in India followed by scheduled caste groups (about 90% of scheduled castes are agricultural laborers) which is followed by caste groups [13].

Average heterozygosity is a measure of genetic diversity at the population scale and indicates the average proportion of individuals that are heterozygous for a given trait. The frequencies of CCVD alleles found in the present study was lower compared to those of the frequencies reported in five populations in Manipur (India) [13] and for science students in the ASC Rahuri College (India) [42]. Furthermore, the level of heterozygosity found in the present study amongst female students of Amhara (8%) and Oromo (8%) ethnic origin was low compared to that of the population in India (e.g. Meitie (26.61%), Sheikh (24.45%) [13]. In addition, the heterozygosity documented in the present study for female students was almost half of that of the prevalence in a general population (14.70%) [43]. Similarly, the frequency of double heterozygosity found in the present study for female subjects was one fifth of the prevalence in the general population [43]. Females who had a recessive allele for deutan on one X chromosome, but a recessive allele for protan on the second X chromosome are called ‘double carriers’ (or compound heterozygote) [43]. Carroll [43] has also examined a female subject who had one deutan son and a protan son, which indicated her identity of double heterozygote/double carrier. Furthermore, the identity of this subject as both deutan and protan was also confirmed by genetic analysis. In the present analysis, we did not explicitly find double carrier subjects, but we do not rule out the possibility of getting such subjects.

In some jobs such as doctors, educational trainers and drivers, colour recognition is essential and hence detection of colour vision deficiency at an early age is useful to avoid certain occupational hazards [2, 13, 21, 22]. However, in Ethiopia, early testing of colour vision deficiency is not common when students join university to study fields like engineering, soil sciences, chemistry, electricity and electronics, etc. Since colour vision deficiency is not a deadly disease, an individual can be a colour vision deficiency without noticing the scene. Unnoticed deficiency may lead to professional inefficiency and some risks. A study conducted by Abebe and Wondmikun [24] on defective colour perception amongst licensed car drivers in Addis Ababa revealed a prevalence of 4.50% of colour vision impairment. Furthermore, the results showed that 31.8% of colour blind subjects had road traffic accidents the past three years prior to the study. They have also found that an examination of colour vision in driving license seekers in Ethiopia does not screen out colour-blind individuals. The likelihood that colour-blind drivers would encounter an accident is about twice as high as amongst non-colour-blind drivers [24]. Therefore, in today’s automated world, when every livelihood is connected with modern technology, analysis of colour vision deficiency is indispensable to mitigate the potential impact of colour vision deficiency [2, 13, 21, 24].

### Limitations

The overall prevalence of CCVD found in the present study was lower compared to the previous studies done in Ethiopia. The prevalence of CCVD was highest amongst males, but relatively low amongst female subjects. There was a clear difference in the prevalence of colour vision deficiency amongst students of various ethnic groups. Many students were unaware of colour vision deficiency itself, except students from health science and medicine. Therefore, proper screening, education and counseling are needed to minimize the influences of colour vision deficiency in the country and could be beneficial to the affected subject in tackling difficulties in everyday work and for proper choice of future profession.

## Conclusion and recommendations 

Anomaloscope should be used for detailed analysis of quantitative and qualitative anomalies in colour perception. However, in this study, we did not use anomaloscope which is clinically used for screening and diagnosis of CVD, because the instrument was not available in our set up. Furthermore, we were not able to cover all the ethnic groups as it was not possible for logistic factors.

## Data Availability

The data used in this study are available from the corresponding author on reasonable request.
